# Current Evidence on Auricular Therapy for Chemotherapy-Induced Nausea and Vomiting in Cancer Patients: A Systematic Review of Randomized Controlled Trials

**DOI:** 10.1155/2014/430796

**Published:** 2014-11-25

**Authors:** Jing-Yu Tan, Alexander Molassiotis, Tao Wang, Lorna K. P. Suen

**Affiliations:** ^1^School of Nursing, The Hong Kong Polytechnic University, Hung Hom, Kowloon, Hong Kong; ^2^The Second Affiliated People's Hospital, Fujian University of Traditional Chinese Medicine, No. 13, Hudong Road, Gulou District, Fuzhou 350003, China

## Abstract

Auricular therapy (AT) has been historically viewed as a convenient approach adjunct to pharmacological therapy for cancer patients with chemotherapy-induced nausea and vomiting (CINV). The aim of this study was to assess the evidence of the therapeutic effect of AT for CINV management in cancer patients. Relevant randomized controlled trials were retrieved from 12 electronic databases without language restrictions. Meanwhile, manual search was conducted for Chinese journals on complementary medicine published within the last five years, and the reference lists of included studies were also checked to identify any possible eligible studies. Twenty-one studies with 1713 participants were included. The effect rate of AT for managing acute CINV ranged from 44.44% to 93.33% in the intervention groups and 15% to 91.67% in the control groups. For delayed CINV, it was 62.96% to 100% and 25% to 100%, respectively. AT seems to be a promising approach in managing CINV. However, the level of evidence was low and the definite effect cannot be concluded as there were significant methodological flaws identified in the analyzed studies. The implications drawn from the 21 studies put some clues for future practice in this area including the need to conduct more rigorously designed randomized controlled trials.

## 1. Introduction

Chemotherapy-induced nausea and vomiting (CINV) is one of the most common and distressing side effects among cancer patients, and the severity and incidence of CINV are usually determined by the particular chemotherapeutic agent, dosage, combinations with other treatment approaches, and patient characteristics [1–3]. Some of the most commonly used regimens, such as cisplatin or cyclophosphamide, are regarded to be of moderate to high emetic potential, respectively [3, 4], which can have a significant negative impact on patients' health status. CINV occurs in approximately 40–80% of cancer patients when receiving chemotherapy with moderate to high emetic potential [5], and it can severely impair the patients' physical and psychological status. Complications caused by CINV may increase unnecessary healthcare costs, aggravate burden on medical and nursing resources, and prolong hospitalizations [6, 7]. Moreover, uncontrolled CINV can also decrease the patients' quality of life, influence their physical activities and social function, and induce emotional problems [8, 9].

The most popular approach applied for controlling CINV nowadays is the regular use of antiemetic drugs. Of which, 5-HT_3_ receptor antagonists, NK-1 receptor antagonists, and corticosteroids are identified as first-line treatment [1]. However, even with the help of antiemetics, nearly half of cancer patients receiving moderate to high emetic chemotherapy still experience some CINV, particularly delayed nausea and vomiting [10]. In addition to their therapeutic effects, antiemetic drugs can also produce some undesirable side effects, such as constipation, dizziness, and headache [11, 12]. Considering the fact that CINV is very difficult to be completely controlled by antiemetic drugs alone, healthcare professionals should explore other nonpharmacological approaches as adjuncts to pharmacological interventions. Nonpharmacological interventions used in CINV refer to a variety of approaches including acupuncture, acupressure, massage, progressive muscle relaxation, exercise, and psychological support [13]. Of which, acupuncture-point stimulation on* neiguan* (P6) is one of the most popular techniques and it is recommended as a “likely to be effective” approach for managing CINV [13]. Despite the fact that the current evidence of acupuncture-point stimulation for CINV is still judged as highly suggestive but not conclusive [13–16], this kind of intervention has already drawn extensive attention in clinical practice and has been accepted by a wide range of healthcare professionals and cancer patients. However, as another therapeutic modality of acupuncture, the role of auricular therapy (AT) has received less attention.

AT is defined as “a health care modality whereby the external surface of the ear, or auricle, is stimulated to alleviate pathological conditions in other parts of the body” [17]. The history of AT can be traced back to more than 2000 years ago in ancient China, and the modern theory and practice of AT was developed by Paul Nogier, a French physician, in the late 1950s [17, 18]. Different from the Chinese body acupuncture/acupressure which is based on the traditional meridian-collateral theory that 12 regular meridians (six* yin* meridians and six* yang* meridians) run throughout the body with a number of acupuncture points distributed along their paths [17], AT focuses on the connection between the specific acupoints located in the auricle and pathological conditions in particular* zang-fu* organs of the body [17, 18]. The modern theory of AT recognizes that the external ear has a somatotopic pattern with an inverted fetus within the uterus, with the head and facial areas towards the bottom of the auricle and the feet areas towards the upper rim of the auricle [17, 18]. Each part of the body corresponds to an auricular acupoint or a specific area which reflects the pathological and/or physiological condition [17, 18]. By stimulating sensitive auricular acupoints which correspond to particular* zang-fu* organs or specific parts of the body, AT could generate a positive impact on treating or relieving a variety of health problems.

Clinical studies and systematic reviews have provided some evidence that AT can effectively control hypertension, anxiety, and pain intensity, as well as reducing weight, and the WHO has recognized it as a promising therapeutic approach for its effectiveness in managing a variety of disorders [18–22]. Theoretically, by stimulating auricular acupoints which are closely related to human body's gastrointestinal function, AT could also alleviate various types of gastrointestinal problems such as nausea and vomiting [18]. Many clinical studies were conducted during the past several years to investigate the effectiveness of AT for managing CINV but their methodological quality and clinical findings have not yet been systematically summarized and the overall evidence is still uncertain. To our knowledge, there are no systematic or narrative reviews evaluating the effect of AT in cancer patients who experience CINV. This systematic review aimed to explore the therapeutic outcomes and current level of evidence of AT for CINV among cancer patients, to identify limitations in current practice, and thereby to provide recommendations for future research and practice in this area.

## 2. Methods

### 2.1. Data Sources and Searching Strategies

A study protocol accompanied by a data extraction form was formulated and was critically reviewed by two experts prior to the initiation of this study. Relevant studies were mainly searched and retrieved from electronic databases, and a total of 12 databases (from inception to May 27, 2014) were accessed, which included PubMed, EMBase, Cochrane Central Register of Controlled Trials (CENTRAL), CINAHL, AMED, PsycINFO, Thomson Reuters Web of Science, Science Direct, China National Knowledge Infrastructure (CNKI), WanFang Data, Chinese Scientific Journal Database (VIP), and Chinese Biomedical Literature Database (CBMdisc). No language restriction was applied for electronic searches. Meanwhile, manual search was conducted in Chinese journals of complementary medicine published within the last five years, and the reference lists of final included studies were also checked to identify any possible eligible studies. Two reviewers (Tan J. Y. and Wang T.) independently searched the literature according to the study protocol. MeSH terms, key words, and free words such as “auriculotherapy,” “acupuncture, ear,” “auricular therap∗,” “ear acupunctur∗,” “nausea,” “vomiting,” “antiemetic∗,” “chemotherapy,” “antineoplastic agent∗,” and “neoplasms,” were used in the search strategies.  Table 1 presents the two main search strategies for this study.

### 2.2. Inclusion and Exclusion Criteria

Inclusion criteria for this systematic review were (1) randomized controlled trials, (2) cancer patients with acute or delayed nausea and vomiting after receiving chemotherapy, and (3) trials comparing AT with or without antiemetic medications to one or more of the following: sham AT control, concomitant antiemetic medications, usual care, waiting-list control, or no treatment. Types of AT could be auricular acupressure, auricular manual/electronic/laser acupuncture, auricular moxibustion, auricular injection, or auricular bloodletting therapy, and so forth. Clinical case reports and case series, nonrandomized controlled trials, or other uncontrolled clinical trials were excluded.

### 2.3. Study Selection and Data Extraction

Study characteristics and outcome data of each included trial were extracted using the data extraction form, which included (1) first author, year of publication, study design, and setting, (2) patient and disease characteristics (age, gender, sample size, types of cancer, chemotherapy agents, and antiemetics used), (3) intervention protocols (types of AT, selected auricular acupoints, approaches for acupoint detection, manual pressing instruction, and treatment duration) and types of control, (4) outcome measures and the therapeutic effects of AT for CINV, and (5) possible adverse events associated with AT. Study selection and data extraction were also conducted by two reviewers independently. Any disagreement about the studies between the reviewers was resolved with further assessment of the study with another reviewer, reaching consensus.

### 2.4. Quality Assessment for the Included Studies

The Cochrane collaboration's tool for risk of bias was utilized to evaluate the methodological quality of each trial [23]. The tool consists of seven specific domains: random sequence generation, allocation concealment, blinding of participants and personnel, blinding of outcome assessment, incomplete outcome data, selective outcome reporting, and other bias [23]. In this review, “other bias” was further clarified into several categories to assess whether the author reported sample size calculations, the baseline assessment, the diagnostic, inclusion and exclusion criteria, the evaluation of therapeutic effect, the adverse events, and the method of data analysis in the paper. Each item was rated as “low risk of bias,” “unclear risk of bias,” or “high risk of bias.” Disagreement between reviewers was resolved through discussion.

### 2.5. Outcomes Assessment

Incidence and severity of CINV was the primary outcome of this systematic review, and type and frequency of AT-related adverse events, patients' physical performance status, and emotional conditions (anxiety and depressive symptoms) were set as secondary outcomes. A meta-analysis using the Review Manager 5.2 was originally planned. However, because of the significant methodological flaws identified in the analyzed literature as well as clinical heterogeneity of the types of cancer, intervention protocols, and control methods used among studies, meta-analysis was deemed inappropriate and a descriptive analysis was employed to summarize the therapeutic effect of AT for both the primary and secondary outcomes.

## 3. Results

### 3.1. Characteristics of Included Studies

**Figure 1:**
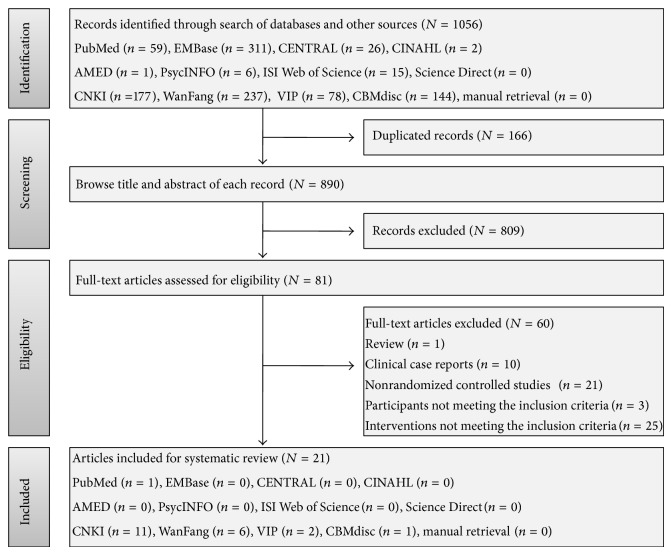
Flow chart of study selection for the systematic review. CENTRAL: Cochrane Central Register of Controlled Trials, CNKI: China National Knowledge Infrastructure, VIP: Chinese Scientific Journal Database, and CBM: Chinese Biomedical Literature Database.

Electronic and manual searches yielded 1056 records. One hundred and sixty-six duplicated items were removed and another 809 items were further excluded after browsing the title and abstract. Full-text articles of the remaining 81 records were retrieved for the assessment of their eligibility, of which, 60 articles were finally excluded because they were not a randomized design (*n* = 21) or were clinical case repots (*n* = 10) or narrative review (*n* = 1), and the participant (*n* = 3) and intervention (*n* = 25) did not meet the inclusion criteria of the systematic review. Therefore, 21 studies [24–44] were identified for analysis. The flow chart of study selection is presented in Figure 1.

The included studies (20 journal articles and one master's thesis) were published between 2003 and 2013, with two English and 19 Chinese articles. One trial [29] was conducted in Taiwan, and the other 20 were carried out in different provinces of Chinese mainland. Studies focused on different types of malignancies, including breast cancer in five studies [28, 33–35, 44], lung cancer in two studies [24, 37], leukemia in two studies [25, 30], gastrointestinal cancer in one study [39], and pediatric cancers in one study [29]. Seven studies [26, 27, 32, 36, 40, 42, 43] included more than two types of cancer, while the other three studies [31, 38, 41] did not specify what kind of cancer patients they included. A total of 1713 participants were identified from the included trials, with the age ranging from 6 to 80 years. All participants were recruited from outpatient clinics or inpatient departments. The average sample size was 81 (range = 10–173), and only eight studies had a sample size of more than 100 subjects [24, 27, 30, 32, 33, 36, 37, 43].

All participants in the intervention groups received AT before and after chemotherapy, of which, 20 studies applied auricular acupressure, while another one [28] chose auricular acupuncture. Of the 20 studies that used auricular acupressure as the intervention approach, 15 [24–26, 30–37, 39–42] investigated the therapeutic effect of auricular acupressure by comparing auricular acupressure plus antiemetic drugs with antiemetic drugs alone and two [29, 38] incorporated a sham acupressure control group (stimulation of nonspecific auricular acupoints which are not related to CINV) comparing data from it with the intervention group using the same antiemetics. Furthermore, intervention groups in another two studies [43, 44] only received auricular acupressure while the control groups were treated with antiemetic drugs, and the other study [27] used “usual care” to describe the conventional medical care in both groups but failed to specify whether they have included an antiemetic treatment.

The primary outcome was the incidence and severity of CINV in 20 studies, and only one [39] used different dosages of antiemetic drugs received between groups to evaluate the therapeutic effect of AT. The secondary outcomes in the analyzed trials included physical performance status, adverse events associated with AT or antiemetics, incidence of constipation and abdominal bloating, anxiety, and depressive symptoms. Although reports of adverse events are an important issue to evaluate the safety of AT, there were only three studies [29, 31, 40] that stated such events. Basic characteristics of the analyzed studies can be seen in Table 2.

### 3.2. Description of Auricular Therapy Protocols

A summary of included AT protocols is presented in Table 3. All studies briefly described the AT protocols, which included the selection and identification of targeted auricular acupoints, instructions on manual pressing, and duration of treatment. Of the studies that employed auricular acupressure as therapeutic approaches,* Vaccaria* seeds were the most commonly applied pellets (16 studies), while magnetic pellets (one study) and radish seeds (one study) were also used for acupressure. The number of selected acupoints ranged from three to eleven, with three to seven main acupoints and two to four adjunct points. Among which,* shenmen* (21 studies) and stomach (21 studies) were the most commonly selected main acupoints, followed by sympathetic (14 studies), spleen (12 studies), liver (10 studies), subcortex (8 studies), cardia (6 studies), and so forth, which were applied as additional main or adjunct acupoints.

Fourteen studies described the methods used for acupoint identification, and the acupoint detector, an electronic finder, was used in 10 studies, and cotton swab or needle was used in four studies, whereas the other seven studies did not specify the method for acupoint identification. Instructions on manual pressing of taped pellets varied. The frequencies of manual pressing ranged from three to eight times a day, and the duration of pressing for each acupoint was quite inconsistent among studies. Pressing each acupoint for no more than two minutes was mentioned in six studies [26, 30, 35, 37, 40, 44]; however, there were four studies [31, 38, 42, 43] that required that participants press each acupoint for 3–5 minutes each time. A sensation of* de qi* was described in the majority of the included studies to indicate the sign of therapeutic efficacy.* De qi* is a TCM terminology that illustrates a subjective feeling of numbness, pressure sensation, soreness, or distension induced by acupuncture or acupressure, which is viewed as an immediate indicator of accurate acupoint location and a treatment efficacy [40]. Some studies informed participants to press acupoints before meals and/or before and after chemotherapy, and, in addition to the regular manual pressing, three studies [28, 29, 37] also required participants to press the seeds as soon as they felt nausea and vomiting.

The treatment duration varied significantly among studies. One study [28] only used a one-day AT, one study [38] conducted a two-day treatment, one [27] study utilized AT for three days, and five studies [29–31, 35, 40] applied the intervention for seven days, while nearly half of the included trials provided AT during the whole chemotherapy cycle. Unfortunately, those studies which designed an AT treatment for a complete chemotherapy cycle failed to specify the specific length of each treatment. Apart from these, the longest treatment course was mentioned in one study [32], which was 27 days for one AT treatment over two treatments in total.

### 3.3. Methodological Quality and Risk of Bias for the Included Trials

Table 4 shows the methodological assessment of the included trials. Significant methodological flaws were identified. Randomization was mentioned in all studies, whereas only three [24, 29, 30] described the details of generating the random sequence by a random number table or computer-based randomization. One study [40] did not have blinding for participants, and, for all other studies, there was no sufficient information to judge whether they applied adequate blinding. All of the 21 studies failed to specify whether they conducted allocation concealment. One study [32] reported the dropout rate of subjects but failed to perform an intention-to-treat (ITT) analysis to handle missing data, and two studies [30, 35] selectively reported the study outcomes. In terms of “other bias,” no studies claimed they had calculated sample size to determine how many subjects were appropriate for their study except for one pilot testing [29] which had estimated the effect size of the primary outcome for a future main trial; baseline assessments were conducted in the majority of studies; more than half of the studies clearly described the diagnostic, inclusion, and exclusion criteria for study subjects; all studies described the therapeutic evaluation criteria for the primary or secondary outcomes; only three [29, 31, 40] included adverse events as a secondary outcome, and 18 studies [24, 25, 28–43] had clearly described the statistical methods used for data analysis.

### 3.4. Therapeutic Effect of Auricular Therapy for CINV

Table 5 summarizes the therapeutic effects of AT for CINV. Twenty studies [25–44] specified the criteria used for evaluating the therapeutic effect of AT for CINV. The most common tool utilized was the WHO Recommendations for Grading of Acute and Subacute Toxicity (17 studies). One study [29] employed the Morrow Assessment of Nausea and Emesis (MANE) Scale and one [40] used the National Cancer Institute (NCI) Common Toxicity Criteria, whereas the other one [39] used the different dosages of antiemetics received between groups to evaluate the therapeutic effect of AT. For studies that employed the WHO or the NCI Criteria, therapeutic effects were normally classified into three categories: “markedly effective,” “effective,” and “not effective.” However, the cutoff point for each category was inconsistent among studies, and more than half used grades 0–2 to represent “markedly effective” and “effective,” which means no nausea and vomiting or only experiencing transient vomiting, and grade 3 or above to indicate “not effective,” which means uncontrolled or intractable vomiting requiring additional therapy.

Due to the significant methodological flaws and clinical heterogeneity identified in the included trials, meta-analysis was not conducted and a descriptive analysis was employed to summarize the therapeutic effect of AT for both primary and secondary outcomes. Among all the analyzed trials, the effective rate of AT for managing acute CINV ranged from 44.44% to 93.33% in the intervention groups and from 15% to 91.67% in the control groups. For delayed CINV, it was 62.96% to 100% and 25% to 100%, respectively.

Of the 15 studies [24–26, 30–37, 39–42] that compared auricular acupressure plus antiemetic medications with antiemetic medications alone, the combination of AT and medications was found to be more effective in controlling CINV than using antiemetic drugs alone, with the effective rate ranging from 84.62% to 100% in the intervention groups and from 34.38% to 100% in the control groups. However, there were only four studies [30, 31, 40, 41] separately investigating the therapeutic effect of auricular acupressure for either acute or delayed CINV. Of them, one study [31] reported significantly better outcomes for both acute and delayed CINV in the intervention groups compared with those in the control groups, and one [41] detected a slightly positive trend of AT for managing acute CINV and a significantly better effect for controlling delayed nausea and vomiting. One study [30] found no difference between groups for the management of acute CINV (effective rate: 88% in both groups), and positive effect of AT was only detected for the delayed nausea and vomiting where the effective rate ranged from 90% to 94% in the intervention group and from 70% to 78% in the control group from day 2 to day 7 of the chemotherapy cycle. While the other study [40] only showed that AT plus antiemetic drugs was slightly more effective in relieving acute vomiting than using antiemetic drugs alone.

There was only one study [28] employing auricular acupuncture as adjunct to conventional antiemetic medications, and CINV was also evaluated according to the WHO recommendations. Auricular acupuncture combined with antiemetic drugs was reported to be more effective than using antiemetic drugs alone, with the effective rate being 87.50% in the intervention group and 45% in the control group. However, conflicting results were reported for both comparisons of “auricular acupressure versus sham acupressure” [29, 38] and “auricular acupressure alone versus antiemetic drugs alone” [43, 44], where significant differences between groups were stated in two studies [38, 44], while the other two [29, 43] reported no significant differences.

In addition to the primary outcome, some studies also focused on the effect of AT on physical performance status and emotional conditions among cancer patients undergoing chemotherapy. Three studies [36, 37, 40] employed physical performance status as the secondary outcome and showed a favorable effect (as measured by the Karnofsky Performance Scale Index) in the intervention groups compared with that in the control groups. One study [24] evaluated patients' emotional conditions (as measured by the Hamilton Anxiety/Depression Scale) and reported positive effects of AT in relieving patients' anxiety and depressive symptoms. Regarding the adverse events identified in the analyzed literature, one study [29] reported adverse events caused by pellet tapes, which was itching in three cases. In another study [31], the reported adverse events were adverse drug reactions caused by antiemetic medications, such as constipation, headache, and fatigue, and the author compared the incidence of these events between groups and stated that AT can also relieve adverse drug reactions.

## 4. Discussion

Encouraging results of AT for CINV management were reported in our analyzed studies. However, because the methodological quality of the included trials was generally poor, a definite effect is uncertain and the strength of evidence of AT for preventing and treating CINV is limited and currently not convincing.

According to the theory of traditional Chinese medicine, “dysfunction of spleen in transportation” and “stomach disharmony” are the principal pathogeneses for inducing CINV, and chemotherapy agents are regarded as having the evils of fire toxin and are considered to be of high risk for disturbing gastrointestinal functions [47, 48]. By stimulating the acupoints which reflect the specific parts of body or* zang-fu* organs, AT can activate related meridians, regulate* qi*-blood circulation and* zang-fu* organ functions, and, therefore, achieve a therapeutic effect for various disorders [18, 49, 50].

In our analyzed studies,* shenmen*, stomach, sympathetic, spleen, and liver were the most popular selected AT acupoints for controlling CINV. According to AT theories,* shenmen*, located in the apex of the triangular fossa, is the main acupoint most commonly referred to for controlling nausea and vomiting with the role of tranquilizing the mind, facilitating serenity, and soothing the nerves [18, 51]. Stomach is another main acupoint for treating gastrointestinal disorders which can be found on the medial concha ridge; it has the potential of harmonizing the stomach and rectifying* qi* [51]. Sympathetic is also widely used for managing CINV by reducing hyperresponsiveness of the sympathetic nervous system and alleviating spasm of gastrointestinal smooth muscles [18, 51]. Spleen is internally and externally connected with the stomach and it is involved in all muscular movements, whereas liver is mainly responsible for facilitating the free flow of* qi*. When dealing with CINV, spleen and liver are chosen for strengthening the stomach functions and relieving muscle tension and spasms [18, 51]. When these acupoints are stimulated, the gastric-related meridians would be activated to rebalance* yin* and* yang* between stomach and spleen and produce positive effects on relieving nausea and vomiting [18, 51].

All studies described the AT protocols, and the majority utilized auricular acupressure as the intervention. Compared with other types of AT with needle or moxibustion, auricular acupressure, which employs small-sized round objects such as plant seeds or magnet pellets to press the auricular acupoints, is noninvasive, relatively risk free, and easily administrated [49, 51, 52]. The reference for identification of auricular acupoints was seldom mentioned in these studies except for two [29, 40] which used Huang's ear reflex theory [29] and the Chinese Standard Ear Acupoints Chart [40]. We suggest future studies to use the Chinese Standard Ear Acupoints Chart as the guide for locating and identifying acupoints. Meanwhile, the former widely used Chinese Standard Ear Acupoints Chart, which was recommended by the WHO in 1992, has already been updated in 2008, which is the Chinese Standard Ear Acupoints Chart Nomenclature and Location of Auricular Points (GB/T-13734-2008) [53]. For the frequency of manual pressing, it is pointed out that the manual pressing frequencies are closely related to the therapeutic effect of AT [51]. However, significant inconsistencies on pressing instructions were found among trials. The length of pressing each acupoint ranged from 30 seconds to 5 minutes, while some authors also instructed participants to use the* de qi* sensation as an indicator of stopping the pressure. This contradicting statement would confuse readers, and it should be noted that continuous pressing with inappropriate intensity may also induce some side effects to the ear skin, such as skin breakdown and subsequent infection.

Compliance with treatment is of significant importance as appropriate compliance could be linked with a more desired outcome and a more credible study result [54]. However, 20 of the included studies failed to monitor patients' compliance with AT and the incomplete compliance or noncompliance could produce detrimental effects for the outcome assessment and lead to type II errors [54, 55]. Because various types of cancers were included, the treatment duration varied significantly among studies. More than half stated that the AT covered the present chemotherapy cycle, but this ambiguous description made unclear the exact length (days) for each treatment course. Meanwhile, some trials only utilized one or two days of intervention, which seems quite insufficient for investigating the effect of AT for delayed CINV, as the delayed phase typically lasts for five to seven days (or more) after chemotherapy.

The majority of included studies reported positive CINV outcomes through the use of AT. However, it is worth noting that there were only six studies [29–31, 38, 40, 41] that separately investigated the effects of AT on either acute or delayed CINV, and outcomes were seldom reported for nausea and vomiting separately. The overall impact as well as the particular impact of AT on both acute and delayed CINV should be investigated further, particularly with regard to delayed CINV which is more difficult to be managed with current antiemetics. Also, the instrument used for outcome evaluation in most included studies was the WHO Recommendations for Grading of Acute and Subacute Toxicity. The scale was developed in the late 1970s and the assessment criteria were mainly based on healthcare provider's observations [45]. A lack of clear description of the severity and incidence of nausea or vomiting for each grade limits the applicability and reliability of the tool used in clinical practice, and detection bias may also be unavoidable as the evaluation is mainly dependent on researchers' subjective observations.

One of the advantages of complementary therapies may be being relatively free of risks, which means less adverse events. Reporting adverse events is an important issue in clinical studies; however, only two studies [29, 40] included “adverse events associated with AT” as outcome measures, and this review cannot prove the safety of AT because the sample size included in the studies which reported adverse events was too small.

Although encouraging results of AT for CINV management were reported, the strength of evidence was still rated as low because of the significant methodological flaws identified in the analyzed studies. Studies with unsatisfactory methodological quality are more inclined to overestimating the effect size [56]. Description of random sequence generation was absent in most of the included studies, and the blinding design was unclear in all studies. Even if it is difficult to conduct blinding for participants in studies that compare AT with antiemetic drugs, a blinding design of the outcome assessment and data analysis is possible, and it is pointed out that incomplete blinding in RCTs can exaggerate the observed effect size [57]. All of these methodological flaws could affect the study results and make the current evidence on AT for CINV management of low quality and limited value.

There are some limitations for this review. Although we have made efforts to retrieve all relevant studies, the articles identified for analysis were only English and Chinese publications. Japanese and Korean databases failed to be accessed and language bias may be possible. Since the reviewers are only able to read Chinese and English, coinvestigators who are familiar with other languages should be considered in future studies. At the same time, we cannot exclude the possibility of publication bias because negative outcomes were seldom reported in our included studies, and the majority of analyzed studies were only conducted in Chinese mainland.

## 5. Implications for Future Research and Practice

There are some implications from this review. Firstly, a detailed AT protocol should be formulated which clearly describes the identification of main and/or adjunct acupoints, the instruction of manual pressing, and the length of treatment. Selection of the main acupoints should be based on the involved* zang-fu* organs and particular body location of the targeted disorder, while the choice of adjunct acupoints is not essential and should depend on whether the subjects have any accompanying symptoms or depend on the secondary outcomes set in the study. Secondly, more reliable and valid instruments for CINV assessment such as the Functional Living Index Emesis (FLIE), MANE, MASCC Antiemesis Tool (MAT), or Index of Nausea, Vomiting, and Retching (INVR) should be considered in future studies [58]. Thirdly, the treatment duration should be sufficient enough for detecting the effects of AT for both acute and delayed CINV, and patients' compliance with AT should be monitored to give indications of the data quality and to optimize the treatment effect. In addition to emphasizing the therapeutic effect of gastrointestinal symptoms, patients' social and psychological well-being also need attention, and a follow-up approach should be used to monitor the long-term impact of AT on patients' quality of life and emotional status. Moreover, adverse events associated with AT and the likelihood of causality should be recorded and analyzed. Lastly and perhaps most importantly, the methodological quality of future studies needs to be improved, with more clear descriptions of the generation of random sequence and allocation concealment, a reasonable blinding design, and an appropriate method for sample size calculation and effect size estimation, and so forth. Following the STRICTA guidelines for reporting acupuncture trials should be used for the design, description, and reporting of the study methodology.

## Figures and Tables

**Table 1:** Selected searching strategies for the systematic review.

ID	Searching strategies	Records
PubMed		

Number 1	“auriculotherapy”[MeSH Terms] OR “acupuncture, ear”[MeSH Terms]	264

Number 2	((((((((((((((((((auriculotherap^*^[Title/Abstract]) OR (acupunctur^*^[Title/Abstract] AND ear^*^[Title/Abstract])) OR (acupunctur^*^[Title/Abstract] AND auricu^*^[Title/Abstract])) OR (acupressur^*^[Title/Abstract] AND ear^*^[Title/Abstract])) OR (acupressur^*^[Title/Abstract] AND auricu^*^[Title/Abstract])) OR (auricu^*^[Title/Abstract] AND poin^*^[Title/Abstract])) OR (ear[Title/Abstract] AND poin^*^[Title/Abstract])) OR (ear[Title/Abstract] AND acupoin^*^[Title/Abstract])) OR (auricu^*^[Title/Abstract] AND plaster^*^[Title/Abstract])) OR (massag^*^[Title/Abstract] AND ear^*^[Title/Abstract])) OR (ear[Title/Abstract] AND plaster^*^[Title/Abstract])) OR (massag^*^[Title/Abstract] AND auricu^*^[Title/Abstract])) OR (magne^*^[Title/Abstract] AND ear^*^[Title/Abstract])) OR (magne^*^[Title/Abstract] AND auricu^*^[Title/Abstract])) OR otopoin^*^[Title/Abstract]) OR (ear[Title/Abstract] AND hol^*^[Title/Abstract])) OR vaccaria^*^[Title/Abstract]) OR seed^*^[Title/Abstract]) OR erxue[Title/Abstract]	136112

Number 3	#1 OR #2	136155

Number 4	((“nausea”[MeSH Terms] OR “vomiting”[MeSH Terms]) OR “drug related side effects and adverse reactions”[MeSH Terms]) OR “antiemetics”[MeSH Terms]	119358

Number 5	(((((((((((((nausea[Title/Abstract]) OR vomiting[Title/Abstract]) OR emesis[Title/Abstract]) OR antiemetic^*^[Title/Abstract]) OR anti-emetic^*^[Title/Abstract]) OR (anti[Title/Abstract] AND emetic^*^[Title/Abstract])) OR puke^*^[Title/Abstract]) OR (gastrointest^*^[Title/Abstract] AND toxicit^*^[Title/Abstract])) OR (gastrointest^*^[Title/Abstract] AND reactio^*^[Title/Abstract])) OR (intest^*^[Title/Abstract] AND toxicit^*^[Title/Abstract])) OR (intest^*^[Title/Abstract] AND reactio^*^[Title/Abstract])) OR (drug[Title/Abstract] AND toxicit^*^[Title/Abstract])) OR (adverse[Title/Abstract] AND reactio^*^[Title/Abstract] AND drug[Title/Abstract])) OR (adverse[Title/Abstract] AND event^*^[Title/Abstract] AND drug[Title/Abstract])	165973

Number 6	#4 OR #5	258892

Number 7	((“neoplasms”[MeSH Terms] OR “drug therapy”[MeSH Terms]) OR “antineoplastic agents”[MeSH Terms]) OR “antineoplastic combined chemotherapy protocols”[MeSH Terms]	3413127

Number 8	(((((((((((neoplasm^*^[Title/Abstract]) OR tumo^*^[Title/Abstract]) OR tumou^*^[Title/Abstract]) OR neoplasia[Title/Abstract]) OR cance^*^[Title/Abstract]) OR carcinom^*^[Title/Abstract]) OR drug therap^*^[Title/Abstract]) OR chemo^*^[Title/Abstract]) OR antineoplasti^*^[Title/Abstract]) OR cytotoxic^*^[Title/Abstract]) OR malignant[Title/Abstract]) OR Oncolog^*^[Title/Abstract]	2432449

Number 9	#7 OR #8	4107165

Number 10	#3 AND #6 AND #9	380

Number 11	((((((((“randomized controlled trial”[Publication Type]) OR “controlled clinical trial”[Publication Type]) OR “ramdomized”[Title/Abstract]) OR “ramdomised”[Title/Abstract]) OR “placebo”[Title/Abstract]) OR “sham”[Title/Abstract]) OR “randomly”[Title/Abstract]) OR “trial”[Title/Abstract]) OR “groups”[Title/Abstract]	2001185

Number 12	(animals[MeSH Terms] NOT (humans[MeSH Terms] AND animals[MeSH Terms]))	3891501

Number 13	#11 NOT #12	1645594

Number 14	#10 AND #13	59

EMBase		

Number 1	‘acupuncture'/exp	35349

Number 2	auriculotherap^*^:ab,ti OR (ear NEAR/3 acupuncture^*^):ab,ti OR (auricu^*^ NEAR/3 acupunctur^*^):ab,ti OR (ear NEAR/3 acupressur^*^):ab,ti OR (auricu^*^NEAR/3 acupressur^*^):ab,ti OR (auricu^*^ NEAR/3 poin^*^):ab,ti OR ‘auricular plaster':ab,ti OR (ear NEAR/3 plaster^*^):ab,ti OR (ear NEAR/3 poin^*^):ab,ti OR (ear NEAR/3 acupoint^*^):ab,ti OR otopoin^*^:ab,ti OR earhole^*^:ab,ti OR (vaccaria^*^ NEAR/15 ear^*^):ab,ti OR (vaccaria^*^ NEAR/15 auricu^*^):ab,ti OR (massag^*^ NEAR/3 auricu^*^):ab,ti OR (massag^*^ NEAR/3 ear^*^):ab,ti OR (cowherb NEAR/15 ear^*^):ab,ti OR (cowherb NEAR/15 auricu^*^):ab,ti OR (seed^*^NEAR/15 auricu^*^):ab,ti OR (seed^*^ NEAR/15 ear):ab,ti OR (magne^*^ NEAR/15 ear^*^):ab,ti OR (magne^*^ NEAR/15 auricu^*^):ab,ti OR erxue^*^:ab,ti	9841

Number 3	#1 OR #2	44116

Number 4	‘chemotherapy induced nausea and vomiting'/exp	2041

Number 5	‘antiemetic agent'/exp	158782

Number 6	‘adverse drug reaction'/exp	1269406

Number 7	‘chemotherapy induced emesis'/exp	5848

Number 8	‘gastrointestinal toxicity'/exp	29236

Number 9	‘chemotherapy induced nausea and vomiting':ab,ti OR nausea:ab,ti OR vomiting:ab,ti OR emesis:ab,ti OR (anti NEAR/3 emetic^*^):ab,ti OR puck^*^:ab,ti OR (gastrointest^*^ NEAR/5 toxicit^*^):ab,ti OR (gastrointest^*^ NEAR/5 reactio^*^):ab,ti OR (intest^*^ NEAR/5 toxicit^*^):ab,ti OR (intest^*^ NEAR/5reactio^*^):ab,ti OR (drug NEAR/10 toxicit^*^):ab,ti OR (adverse NEAR/5 reactio^*^):ab,ti OR (adverse NEAR/5 event^*^):ab,ti	292493

Number 10	#4 OR #5 OR #6 OR #7 OR #8 OR #9	1542758

Number 11	‘neoplasm'/exp	3365367

Number 12	‘chemotherapy'/exp	617924

Number 13	‘antineoplastic agent'/exp	1460316

Number 14	neoplasm^*^:ab,ti OR tumo^*^:ab,ti OR tumou^*^:ab,ti OR neoplasia:ab,ti OR cance^*^:ab,ti OR carcinom^*^:ab,ti OR chemo^*^:ab,ti OR antineoplasti^*^:ab,ti ORcytotoxic^*^:ab,ti OR malignant:ab,ti OR Oncolog^*^:ab,ti	483073

Number 15	#11 OR #12 OR #13 OR #14	4278022

Number 16	#3 AND #10 AND #15	1347

Number 17	‘controlled clinical trial'/exp OR ‘single blind procedure'/exp OR ‘double-blind procedure'/exp OR ‘crossover procedure'/exp	500727

Number 18	random^*^:ab,ti OR crossover^*^:ab,ti OR (cross NEAR/3 over^*^):ab,ti OR placebo:ab,ti OR (doubl^*^ NEAR/3 blind^*^):ab,ti OR (doubl^*^ NEAR/3 mask^*^):ab,ti OR (singl^*^ NEAR/3 blind^*^):ab,ti OR (singl^*^ NEAR/3 mask^*^):ab,ti OR (trebl^*^ NEAR/3 blind^*^):ab,ti OR (trebl^*^ NEAR/3 mask^*^):ab,ti OR (tripl^*^ NEAR/3 blind^*^):ab,ti OR (tripl^*^ NEAR/3 mask^*^):ab,ti OR assign^*^:ab,ti OR allocat^*^:ab,ti OR volunteer^*^:ab,ti	1330046

Number 19	#17 OR #18	1496159

Number 20	‘animal'/exp OR ‘nonhuman'/exp OR ‘animal experiment'/exp	20373542

Number 21	‘human'/exp	15509570

Number 22	#20 AND #21	15208394

Number 23	#20 NOT 22	5165148

Number 24	#19 NOT #23	1327481

Number 25	#16 AND #24	311

**Table 2:** Characteristics of included trials.

Study and setting	Types of cancer	Participants	Chemotherapy agents	Intervention	Control	Outcomes
**S** ^ a^ **1: You et al. 2013 **[24] RCT, Zhejiang Cancer Hospital, Hangzhou, China	Nonsmall cell lung cancer	**Randomized** = 120, **completed** = 120, **intervention group**: 60, **control group**: 60	Etoposide + cisplatin	Auricular acupressure + antiemetic medications (not specified)	Antiemetic medications (not specified)	(i) CINV (ii) Abdominal bloating (iii) Abdominal pain (iv) Blood glucose and insulin (v) Anxiety (vi) Depression (vii) Pain intensity

**S2: Yang and Liang 2013 **[25] RCT, The Second Affiliated Hospital of SUCM, Xianyang, China	Leukemia	**Randomized** = 58, **completed** = 58, M/F = 23/35, age (yr) = 20–67 **intervention group**: 38, **control group**: 20	Cytarabine, homoharringtonine, daunorubicin, cyclophosphamide, mitoxantrone	Auricular acupressure + antiemetic medication (granisetron, 3 mg, IV, qd)	Antiemetic medication (granisetron, 3 mg, IV, qd)	CINV

**S3: Li 2013 **[26] RCT, The first Affiliated Hospital of Soochow University, Suzhou, China	Lung cancer Breast cancer Ovarian cancer Cervical cancer Esophagus cancer Nasopharynx cancer	**Randomized** = 32, **completed** = 32, **intervention group**: 16, M/F = 5/11, age (yr) = 37–65, **control group**: 16, M/F = 4/12, age (yr) = 36–67	Combined chemotherapy based on cisplatin or Pharmorubicin	Auricular Acupressure + antiemetic medications (azasetron, 10 mg, IV, qd + dexamethasone, 10 mg, IV, qd)	Antiemetic medications (azasetron, 10 mg, IV, qd + dexamethasone, 10 mg, IV, qd)	CINV

**S4: Fang 2013 **[27] RCT, Jiangyan TCM Hospital, Taizhou, China	Lung cancer Liver cancer Breast cancer Colon cancer Esophagus cancer Nasopharynx cancer	**Randomized** = 100, **completed** = 100, M/F = 42/58, age (yr) = 35–75 **intervention group**: 50, **control group**: 50	Not reported	Auricular acupressure + usual care	Usual care	CINV

**S5: Zhang et al. 2013 **[28] RCT, Hangzhou TCM Hospital, Hangzhou, China	Breast cancer	**Randomized** = 80, **completed** = 80, **intervention group**: 40, age (yr) = 29–65 (mean: 47) **control group**: 40, age (yr) = 32–67 (Mean: 48)	Not reported	Auricular acupuncture + antiemetic medications (tropisetron, 5 mg, IV, qd)	Antiemetic medications (tropisetron, 5 mg, IV, qd)	CINV

**S6: Yeh et al. 2012 **[29] RCT (Crossover Design), A Large Children's Hospital, Taiwan	Pediatric cancers	**Randomized** = 10, **completed** = 10, M/F = 6/4, age (yr) = 13.29 ± 3.31 (range from 6 to 18) **Intervention group**: NR **control group**: NR (crossover after 1st treatment)	Cyclophosphamide	Auricular acupressure + antiemetic medications (ondansetron or granisetron + dexamethasone)	Auricular acupressure using sham acupoints + antiemetic medications (ondansetron or granisetron + dexamethasone)	(i) CINV (ii) Adverse events (iii) Booklet used to record intensity and duration of acupressure technique

**S7: Zhang et al. 2012 **[30] RCT, The First Affiliated Hospital of GUCM, Guangzhou, China	Acute leukemia	**Randomized** = 100, **completed** = 100, **intervention group**: 50, M/F = 24/26, age (yr) = 45 ± 14.8 (range from 17 to 64), **control group**: 50, M/F = 23/27, age (yr) = 44 ± 15.2 (range from 14 to 62)	DA combination (daunorubicin + cytarabine) or VP combination (vincristine + prednisone)	Auricular acupressure + antiemetic medications (ondansetron, 8 mg, IV, qd)	Antiemetic medications (ondansetron, 8 mg, IV, qd)	(i) CINV (ii) Food intake

**S8: Jiang 2012 **[31] RCT, Zhejiang Cancer Hospital, Hangzhou, China	Not specified	**Randomized** = 85, **completed** = 85, **intervention group**: 43, M/F = 27/16, age (yr) = 36 to 75, **control group**: 42, M/F = 25/17, age (yr) = 33 to 72	Combined chemotherapy based on cisplatin or Adriamycin	Auricular Acupressure + Antiemetic Medication (Granisetron, 8 mg, IV, qd)	Antiemetic medication (granisetron, 8 mg, IV, qd)	(i) CINV (ii) Adverse events

**S9: Zhong et al. 2012 **[32] RCT (Crossover Design), The First Hospital of Wuhan, Wuhan, China	Lung cancer Breast cancer Gastric cancer Colon cancer Ovarian cancer Cervical cancer Tongue cancer Endometrial cancer Nasopharynx cancer	**Randomized** = 100, **completed** = 94, M/F = 58/42, age (yr) = 17–70 (mean: 52.3) **intervention group**: NR **control group**: NR (crossover after 1st treatment)	GP combination (gemcitabine + cisplatin) or TP combination (docetaxel + cisplatin) or AC combination (Adriamycin + cyclophosphamide) or DF combination (cisplatin + fluorouracil), and so forth.	Auricular acupressure + antiemetic medications (tropisetron, qd + diphenhydramine, qd + dexamethasone, qd)	Antiemetic medications (tropisetron, qd + diphenhydramine, qd + dexamethasone, qd)	(i) CINV (ii) Constipation

**S10: Lu 2012 **[33] RCT, Ningbo TCM Hospital of Zhejiang Province, Ningbo, China	Breast cancer	**Randomized** = 128, **completed** = 128, **intervention group**: 64, age (yr) = 45.10 ± 10.42, **control group**: 64, age (yr) = 52.96 ± 6.11	CAF combination (cyclophosphamide + Adriamycin + fluorouracil) or CMF combination (cyclophosphamide + methotrexate + fluorouracil)	Auricular acupressure + antiemetic medication (granisetron, 3 mg, IV, qd)	Antiemetic medication (granisetron, 3 mg, IV, qd)	CINV

**S11: Huang et al. 2012 **[34] RCT, The First Hospital of Wuhan, Wuhan, China	Breast cancer	**Randomized** = 80, **completed** = 80, age (yr) = 28–65 (mean: 46.5) **intervention group**: 40, **control group**: 40	Anthracycline-based combination	Auricular acupressure + antiemetic medications (tropisetron, 4 mg, IV, bid)	Antiemetic medications (tropisetron, 4 mg, IV, bid)	(i) CINV (ii) Constipation

**S12: Wang 2012 **[35] RCT, Xuzhou TCM Hospital of Jiangsu Province, Xuzhou, China	Breast cancer	**Randomized** = 52, **completed** = 52, age (yr) = 47.4 (mean) **intervention group**: 26, **control group**: 26	Cyclophosphamide or fluorouracil or Adriamycin or taxol or doxorubicin or Navelbine	Auricular acupressure + antiemetic medications (azasetron, 10 mg, IV + omeprazole, 40 mg, IV)	Antiemetic medications (azasetron, 10 mg, IV + omeprazole, 40 mg, IV)	(i) CINV (ii) Constipation (iii) Abdominal bloating (iv) Abdominal pain (v) Headache

**S13: Huang 2011 **[36] RCT, The First Hospital of Shangqiu City, Shangqiu, China	Lung cancer Gastric cancer Colon cancer Breast cancer	**Randomized** = 120, **completed** = 120, M/F = 70/50, age (yr) = 30–76 **intervention group**: 60, **control group**: 60	Not reported	Auricular acupressure + antiemetic medications (azasetron)	Antiemetic medications (azasetron)	(i) CINV (ii) Physical performance status

**S14: Liu and Chen 2011 **[37] RCT, The First Affiliated Hospital of GUCM, Guangzhou, China	Lung cancer	**Randomized** = 127, **completed** = 127, **intervention group**: 85, M/F = 53/32, age (yr) = 56 ± 11.4 (range from 31 to 75), **control group**: 42, M/F = 23/19, age (yr) = 51 ± 10.9 (range from 35 to 75)	Platinum-based chemotherapy	Auricular acupressure + antiemetic medications (granisetron, 3 mg, IV, bid or ondansetron, 8 mg, IV, bid)	Antiemetic medications (granisetron, 3 mg, IV, bid or ondansetron, 8 mg, IV, bid)	(i) CINV (ii) Physical performance status

**S15: Ye et al. 2011 **[38] RCT, The General Hospital of Chinese People's Liberation Army, Beijing, China	Not specified	**Randomized** = 47, **completed** = 47, **intervention group**: 27, M/F = 15/12, age (yr) = 58 ± 12 (range from 33 to 76), **control group**: 20, M/F = 11/9, age (yr) = 57 ± 11 (range from 40 to 78)	Not reported	Auricular acupressure + antiemetic medications (tropisetron, 4 mg, IV, bid)	Auricular acupressure using sham acupoints + antiemetic medications (tropisetron, 4 mg, IV, bid)	(i) CINV (ii) Constipation

**S16: Bi 2011 **[39] RCT, LongHua Hospital Affiliated to SUTCM, Shanghai, China	Gastrointestinal cancer	**Randomized** = 50, **completed** = 50, M/F = 23/27, age (yr) = 45.27 **intervention group**: 25, **control group**: 25	Cisplatin + fluorouracil	Auricular acupressure + antiemetic medications (ondansetron, basic dose: 8 mg, IV, qd)	Antiemetic medications (ondansetron, basic dose: 8 mg, IV, qd)	Use of antiemetic medications

**S17: Luo 2011 **[40] RCT, The First Affiliated Hospital of GUCM, Guangzhou, China	Lung cancer Colon cancer Breast cancer Gastric cancer Ovarian cancer Nasopharynx cancer	**Randomized** = 50, **completed** = 50, age (yr) = 20 to 80 (mean: 52.24 ± 10.75) **Intervention group**: 26, M/F = 12/14, **Control group**: 24, M/F = 9/15	Platinum-based chemotherapy	Auricular acupressure + antiemetic medications (granisetron, 3 mg, IV, bid)	Antiemetic medications (granisetron, 3 mg, IV, bid)	(i) CINV (ii) Adverse events (iii) Clinical symptoms (iv) Physical performance status

**S18: Jing 2007 **[41] RCT, Jiangsu Province Hospital of TCM, Nanjing, China	Not specified	**Randomized** = 54, **completed** = 54, **intervention group**: 30, M/F = 20/10, age (yr) = 47.65 **control group**: 24, M/F = 16/8, age (yr) = 47.59	Platinum-based chemotherapy	Auricular acupressure + antiemetic medications (ondansetron, 8 mg, IV, qd)	Antiemetic medications (ondansetron, 8 mg, IV, qd)	CINV

**S19: Qian et al. 2006 **[42] RCT, LongHua Hospital Affiliated to SUTCM, Shanghai, China	Lung cancer Breast cancer Gastrointestinal cancer	**Randomized** = 67, **completed** = 67, M/F = 35/32, age (yr) = 39–77 **intervention group**: 36, **control group**: 31	Combined chemotherapy based on cisplatin or fluorouracil	Auricular acupressure + antiemetic medications (ondansetron, 8 mg, IV, qd)	Antiemetic medications (ondansetron, 8 mg, IV, qd)	CINV

**S20: Zhang et al. 2003 **[43] RCT, LongHua Hospital Affiliated to SUTCM, Shanghai, China	Respiratory cancer Gastrointestinal cancer	**Randomized** = 173, **completed** = 173, **intervention group**: 70, M/F = 28/42, age (yr) = 25–78, **control group 1**: 50, M/F = 19/31, age (yr) = 24–77, **control group 2**: 53, M/F = 23/30, age (yr) = 26–76	MVP combination (mitomycin + vindesine + cisplatin) or NP combination (Navelbine + cisplatin) or FAM combination (fluorouracil + Adriamycin + mitomycin), and so forth.	Auricular acupressure	Control group 1: antiemetic medications (ondansetron, 8 mg, IV, qd) Control group 2: antiemetic medications (metoclopramide, 20 mg, IM, qd)	CINV

**S21: Sun 2003 **[44] RCT, The Affiliated Hospital of SDUTCM, Jinan, China	Breast cancer	**Randomized** = 80, **completed** = 80, **intervention group**: 40, age (yr) = 41.5, **control group**: 40, age (yr) = 42.3	Not reported	Auricular acupressure	Antiemetic medications (metoclopramide, 30 mg, IV)	CINV

RCT: randomized controlled trial, CINV: chemotherapy-induced nausea and vomiting, SUCM: Shanxi University of Chinese Medicine, IV: intravenous, GUCM: Guangzhou University of Chinese Medicine, SUTCM: Shanghai University of Traditional Chinese Medicine, and SDUTCM: Shandong University of Traditional Chinese Medicine.

^a^Study.

**Table 3:** Auricular therapy protocols of included trials.

Study	Types of auricular therapy	Selected auricular acupoints (number) main acupoints (M), adjunct acupoints (A)	Acupoints detection	Instructions of manual pressing	Duration of treatment
S^a^1	Auricular acupressure using *Vaccaria* seeds	**4**: stomach, liver, spleen, *shenmen *	Not reported	3–5 times/day for 1-2 minutes/time until *de qi* ^b^	Not reported

S2	Auricular acupressure using *Vaccaria* seeds	**6**: spleen, stomach, liver, *shenmen*, sympathetic, *sanjiao *	Acupoint detector	5-6 times/day for 2-3 minutes/acupoint/time until *de qi *	A complete chemotherapy cycle

S3	Auricular acupressure using magnetic pellets	**M (7)**: stomach, cardia, esophagus, sympathetic, *shenmen*, spleen, liver **A (4)**: lung, pancreas, gallbladder, adrenal gland	Acupoint detector	Several times/day for 1-2 minutes acupoint/time until *de qi *	Not reported

S4	Auricular acupressure using plant seeds	**4**: stomach, *shenmen*, sympathetic, adrenal gland	Not reported	3-4 times/day for 3 minutes/time	3 days

S5	Auricular acupuncture	**M (3)**: stomach, *shenmen*, sympathetic **A (2)**: liver, spleen	Acupoint detector	4-5 times/day for 1 minute/time, Press when feeling nausea or vomiting	1 day

S6	Auricular acupressure using plant seeds	**5**: *shenmen*, sympathetic, cardia, stomach, digestive subcortex	Acupoint detector	At least 3 times/day for at least 3 periods of 3-minute duration, Press as soon as feeling nausea	7 days

S7	Auricular acupressure using *Vaccaria* seeds	**M (3)**: stomach, sympathetic, *shenmen* **A (4)**: liver, spleen, cardia, esophagus	Not reported	5-6 times/day for 2 minutes/acupoint/time until *de qi *	7 days

S8	Auricular acupressure using radish seeds	**5**: *shenmen*, stomach, sympathetic, spleen, large intestine	Acupoint detector	4 times/day for 5 minutes/acupoint/time until *de qi *	7 days

S9	Auricular acupressure using *Vaccaria* seeds	**7**: stomach, liver, spleen, cardia, *shenmen*, sympathetic, subcortex	Not reported	3 times (morning, noon, night)/day for 5 minutes/time until *de qi *	27 days for one treatment, 2 treatments in total (crossover after 1st intervention)

S10	Auricular acupressure using *Vaccaria* seeds	**4**: stomach, *shenmen*, sympathetic, endocrine	Acupoint detector	Regularly press until *de qi *	Not reported

S11	Auricular acupressure using *Vaccaria* seeds	**M (3)**: stomach, sympathetic, *shenmen* **A (3)**: liver, spleen, large intestine	Not reported	3-4 times/day for 2 minutes/time until *de qi *	A complete chemotherapy cycle

S12	Auricular acupressure using *Vaccaria* seeds	**7**: endocrine, *shenmen*, sympathetic, stomach, cardia, spleen, *sanjiao *	Acupoint detector	8 times (before and after three meals and chemotherapy)/day for 1 minute/acupoint/time until *de qi *	7 days

S13	Auricular acupressure using *Vaccaria* seeds	**M (5)**: stomach, cardia, esophagus, *shenmen*, small intestine **A (2)**: large intestine, rectum	Acupoint detector	4–6 times/day for 60–150 seconds/acupoint/time until *de qi *	A complete chemotherapy cycle

S14	Auricular acupressure using *Vaccaria* seeds	**7**: lung, spleen, stomach, center of ear, large intestine, *shenmen*, subcortex	Not reported	3 times/day for 1-2 minutes/acupoint/time until *de qi* Press when feeling nausea or vomiting	A complete chemotherapy cycle

S15	Auricular acupressure using *Vaccaria* seeds	**M (4)**: stomach, *shenmen*, liver, subcortex **A (4)**: spleen, lung, kidney, heart	Not reported	5-6 times/day for 3–5 minutes/acupoint/time until *de qi *	2 days

S16	Auricular acupressure using *Vaccaria* seeds	**3**: stomach, *shenmen*, subcortex	Acupoint detector	4-5 times/day for 10–15 minutes/time until *de qi *	A complete chemotherapy cycle

S17	Auricular acupressure using *Vaccaria* seeds	**6**: *shenmen*, stomach, sympathetic, subcortex, center of ear, spleen	Cotton swab	3–5 times/day for 30–60 seconds/acupoint/time until *de qi *	7 days

S18	Auricular acupressure using *Vaccaria* seeds	**3**: stomach, *shenmen*, subcortex	Acupoint detector	4-5 times/day for 10–15 minutes/time until *de qi *	From the day prior to the current chemotherapy cycle to two days after the completion of the current cycle

S19	Auricular acupressure using *Vaccaria* seeds	**3**: stomach, *shenmen*, sympathetic	Needle	5-6 times/day for 3–5 minutes/acupoint/time until *de qi *	A complete chemotherapy cycle

S20	Auricular acupressure using *Vaccaria* seeds	**M (3)**: stomach, *shenmen*, sympathetic **A (2)**: liver, subcortex	Needle	5-6 times/day for 3–5 minutes/acupoint/time until *de qi *	A complete chemotherapy cycle

S21	Auricular acupressure using *Vaccaria* seeds	**M (4)**: stomach, *shenmen*, subcortex, center of ear **A (2)**: liver, occiput	Cotton swab	3–5 times/day for 1-2 minutes/acupoint/time until *de qi *	A complete chemotherapy cycle

^a^Study, ^b^ *de qi*: a subjective feeling of numbness, pressure sensation, heaviness, soreness, or distension.

**Table 4:** Methodological quality assessment of included trials.

Criteria	S^a^1	S2	S3	S4	S5	S6	S7	S8	S9	S10	S11	S12	S13	S14	S15	S16	S17	S18	S19	S20	S21
Random sequence generation	✓	?	?	?	?	✓	✓	?	?	?	?	?	?	?	?	?	?	?	?	?	?
Allocation concealment	?	?	?	?	?	?	?	?	?	?	?	?	?	?	?	?	?	?	?	?	?
Blinding of participants and personnel	?	?	?	?	?	?	?	?	?	?	?	?	?	?	?	?	*✗*	?	?	?	?
Blinding of outcome assessment	?	?	?	?	?	?	?	?	?	?	?	?	?	?	?	?	?	?	?	?	?
Incomplete outcome data	?	?	?	?	?	?	?	?	*✗*	?	?	?	?	?	?	?	?	?	?	?	?
Selective outcome reporting	✓	✓	✓	✓	✓	✓	*✗*	✓	✓	✓	✓	*✗*	✓	✓	✓	✓	✓	✓	✓	✓	✓
Other bias	*✗*	*✗*	*✗*	*✗*	*✗*	*✗*	*✗*	*✗*	*✗*	*✗*	*✗*	*✗*	*✗*	*✗*	*✗*	*✗*	*✗*	*✗*	*✗*	*✗*	*✗*
Sample size calculation	*✗*	*✗*	*✗*	*✗*	*✗*	NA^b^	*✗*	*✗*	*✗*	*✗*	*✗*	*✗*	*✗*	*✗*	*✗*	*✗*	*✗*	*✗*	*✗*	*✗*	*✗*
Baseline assessment	✓	✓	✓	✓	✓	✓	✓	✓	*✗*	✓	✓	✓	✓	✓	✓	✓	✓	✓	✓	✓	✓
Diagnostic criteria	✓	✓	✓	✓	*✗*	*✗*	✓	✓	✓	✓	*✗*	*✗*	✓	✓	✓	*✗*	✓	*✗*	✓	✓	*✗*
Inclusion criteria	✓	✓	✓	✓	✓	✓	✓	✓	✓	*✗*	*✗*	*✗*	*✗*	✓	✓	*✗*	✓	*✗*	*✗*	✓	*✗*
Exclusion criteria	*✗*	✓	✓	✓	✓	*✗*	✓	*✗*	✓	✓	*✗*	*✗*	✓	*✗*	✓	*✗*	✓	*✗*	*✗*	*✗*	✓
Evaluation of therapeutic effect	✓	✓	✓	✓	✓	✓	✓	✓	✓	✓	✓	✓	✓	✓	✓	✓	✓	✓	✓	✓	✓
Report of adverse events	*✗*	*✗*	*✗*	*✗*	*✗*	✓	*✗*	✓	*✗*	*✗*	*✗*	*✗*	*✗*	*✗*	*✗*	*✗*	✓	*✗*	*✗*	*✗*	*✗*
Method of data analysis	✓	✓	*✗*	*✗*	✓	✓	✓	✓	✓	✓	✓	✓	✓	✓	✓	✓	✓	✓	✓	✓	*✗*

Based on Cochrane Handbook for Systematic Reviews of Intervention, Part 2: 8.5.

^a^S: study, ✓: low risk of bias; *✗*: high risk of bias; ?: unclear risk of bias.

^b^NA: not applicable due to the design of pilot study.

**Table 5:** Therapeutic effects for CINV and reports of adverse events in included trials.

Study	Evaluation of therapeutic effect	Therapeutic effects			Effective rate	Adverse event
		Markedly effective (number)	Effective (number)	Not effective (number)		
S^a^1	Not reported	Not reported	Nausea: I = 53 (88.33%), C = 47 (78.33%) Vomiting: I = 55 (91.67%), C = 48 (80.00%)	Nausea: I = 7 (11.67%), C = 13 (21.67%) Vomiting: I = 5 (8.33%), C = 12 (20.00%)	Nausea: I = 88.33%, C = 78.33% Vomiting: I = 91.67%, C = 80.00%	Not reported

S2	WHO Recommendations for Grading of Acute and Subacute Toxicity (nausea/vomiting, grades 0–4)^b^	Grade 0 I = 3 (7.89%), C = 1 (5.00%)	Grades 1 I = 34 (89.47%), C = 15 (75.00%)	Grades 2–4 I = 1 (2.63%), C = 4 (20.00%)	I = 97.37%, C = 80.00%	Not reported

S3	WHO Recommendations for Grading of Acute and Subacute Toxicity (nausea/vomiting, grades 0–4)	Grades 0-1 I = 11 (68.75%), C = 7 (43.75%)	Grades 2 1 = 5 (31.25%), C = 6 (37.50%)	Grades 3-4 I = 0 (0.00%), C = 3 (18.75%)	I = 100.00%, C = 81.25%	Not reported

S4	WHO Recommendations for Grading of Acute and Subacute Toxicity (nausea/vomiting, grades 0–4)	Grades 0-1 I = 38 (76.00%), C = 12 (24.00%)	Grades 2-3 I = 7 (14.00%), C = 18 (36.00%)	Grades 4 I = 5 (10.00%), C = 20 (40.00%)	I = 90.00%, C = 60.00%	Not reported

S5	WHO Recommendations for Grading of Acute and Subacute Toxicity (nausea/vomiting, grades 0–4)	Grade 0 I = 13 (32.50%), C = 7 (17.50%)	Grades 1-2 I = 22 (55.00%), C = 11 (27.50%)	Grades 3-4 I = 5 (12.50%), C = 22 (55.00%)	I = 87.50%, C = 45.00%	Not reported

S6	Morrow assessment of nausea and emetics^c^	Real auricular acupressure group showed a better impact on managing CINV compared with usual care group (*P* < 0.05); no difference in CINV can be found between real acupressure group and sham acupressure group.			Not applicable	Itching of the tapes (*n* = 3)

S7	WHO Recommendations for Grading of Acute and Subacute Toxicity (nausea/vomiting, grades 0–4), Diagnostic standard for TCM differentiation of symptoms and signs^d^	Grades 0-1 **Day 1: acute CINV** I = 12 (24.00%), C = 12 (24.00%) **Day 2–7: delayed CINV** Day 2: I = 12 (24.00%), C = 8 (16.00%) Day 3: I = 13 (26.00%), C = 10 (20.00%) Day 4: I = 13 (26.00%), C = 10 (20.00%) Day 5: I = 12 (24.00%), C = 8 (16.00%) Day 6: I = 13 (26.00%), C = 10 (20.00%) Day 7: I = 13 (26.00%), C = 10 (20.00%)	Grade 2 **Day 1: acute CINV** I = 32 (64.00%), C = 32 (64.00%) **Day 2–7: delayed CINV** Day 2: I = 34 (68.00%), C = 30 (60.00%) Day 3: I = 33 (66.00%), C = 29 (58.00%) Day 4: I = 33 (66.00%), C = 29 (58.00%) Day 5: I = 33 (66.00%), C = 28 (56.00%) Day 6: I = 34 (68.00%), C = 25 (50.00%) Day 7: I = 34 (68.00%), C = 26 (52.00%)	Grades 3-4 **Day 1: acute CINV** I = 6 (12.00%), C = 6 (12.00%) **Day 2–7: delayed CINV** Day 2: I = 4 (8.00%), C = 12 (24.00%) Day 3: I = 4 (8.00%), C = 11 (22.00%) Day 4: I = 4 (8.00%), C = 11 (22.00%) Day 5: I = 5 (10.00%), C = 14 (28.00%) Day 6: I = 3 (6.00%), C = 15 (30.00%) Day 7: I = 3 (6.00%), C = 14 (28.00%)	**Day 1: acute CINV** I = 88.00%, C = 88.00% **Day 2–7: delayed CINV** Day 2: I = 92.00%, C = 76.00% Day 3: I = 92.00%, C = 78.00% Day 4: I = 92.00%, C = 78.00% Day 5: I = 90.00%, C = 72.00% Day 6: I = 94.00%, C = 70.00% Day 7: I = 94.00%, C = 72.00%	Not reported

S8	WHO Recommendations for Grading of Acute and Subacute Toxicity (nausea/vomiting, grades 0–4)	Grade 0 **Day 1: acute CINV** I = 0 (00.00%), C = 0 (00.00%) **Day 2–7: delayed CINV** I = 0 (00.00%), C = 0 (00.00%)	Grades 1-2 **Day 1: acute CINV** I = 39 (90.70%), C = 29 (69.05%) **Day 2–7: delayed CINV** I = 43 (100.00%), C=36 (85.71%)	Grades 3-4 **Day 1: acute CINV** I = 4 (9.30%), C = 13 (30.95%) **Day 2–7: delayed CINV** I = 0 (00.00%), C = 6 (14.29%)	**Day 1: acute CINV** I = 90.70%, C = 69.05% **Day 2–7: delayed CINV** I = 100.00%, C = 85.71%	I: fatigue (*n* = 1), constipation (*n* = 11) C: headache (*n* = 2), constipation (*n* = 20), fatigue (*n* = 5)^e^

S9	WHO Recommendations for Grading of Acute and Subacute Toxicity (nausea/vomiting, grades 0–4)	Grade 0 I = 44 (46.81%), C = 23 (24.47%)	Grades 1-2 I = 45 (47.87%), C=58 (61.70%)	Grades 3-4 I = 5 (5.32%), C = 13 (13.83%)	I = 94.68%, C = 86.17%	Not reported

S10	WHO Recommendations for Grading of Acute and Subacute Toxicity (nausea/vomiting, grades 0–4)	Complete relief (grade 0) I = 28 (43.75%), C = 4 (6.25%)	Partial relief (Grade 1-2) I = 32 (50.00%), C=18 (28.13%)	Minor relief + failure (grades 3-4) I = 4 (6.25%), C = 42 (65.63%)	I = 93.75%, C = 34.38%	Not reported

S11	WHO Recommendations for Grading of Acute and Subacute Toxicity (nausea/vomiting, grades 0–4)	Grades 0-1 I = 28 (70.00%), C = 23 (57.50%)	Grade 2 I = 10 (25.00%), C = 9 (22.50%)	Grades 3-4 I = 2 (5.00%), C = 8 (20.00%)	I = 95.00%, C = 80.00%	Not reported

S12	WHO Recommendations for Grading of Acute and Subacute Toxicity (nausea/vomiting, grades 0–4)	Grades 0-1 I = 19 (73.08%), C = 12 (46.15%)	Grade 2 I = 6 (23.08), C = 9 (34.62%)	Grades 3-4 I = 1 (3.85%), C = 5 (19.23%)	I = 96.15%, C = 80.77%	Not reported

S13	WHO Recommendations for Grading of Acute and Subacute Toxicity (nausea/vomiting, grades 0–4)	Grades 0-1 I = 32 (53.33%), C = 7 (11.67%)	Grade 2 I = 20 (33.33%), C = 24 (40.00%)	Grades 3-4 I = 8 (13.33%), C = 29 (48.33%)	I = 86.67%, C = 51.67%	Not reported

S14	WHO Recommendations for Grading of Acute and Subacute Toxicity (nausea/vomiting, grades 0–4)	Grade 0 I = 16 (18.82), C = 5 (11.90%)	Grade 1 I = 62 (72.94%), C = 27 (64.29%)	Grades 2–4 I = 7 (8.24%), C = 10 (23.81%)	I = 91.76%, C = 76.19%	Not reported

S15	WHO Recommendations for Grading of Acute and Subacute Toxicity (nausea/vomiting, grades 0–4)	Grades 0-1 **Acute CINV** I = 5 (18.52%), C = 1 (5.00%) **Delayed CINV** I = 11 (40.74%), C = 1 (5.00%)	Grade 2 **Acute CINV** I = 7 (25.93%), C = 2 (10.00%) **Delayed CINV** I = 6 (22.22%), C = 4 (20.00%)	Grades 3-4 **Acute CINV** I = 15 (55.56%), C = 17 (85.00%) **Delayed CINV** I = 10 (37.04%), C = 15 (75.00%)	**Acute CINV** I = 44.44%, C = 15.00% **Delayed CINV** I = 62.96%, C = 25.00%	Not reported

S16	Use of antiemetic medications (ondansetron), (categories: >20 mg/d, 10–20 mg/d, and <10 mg/d)	Number of patients using ondansetron at dose of >20 mg/d in intervention group was significantly less than those in control group. Number of patients using ondansetron at dose of <10 mg/d in intervention group was significantly more than those in control group. No statistical difference can be found in the number of patients using ondansetron at dose of 10–20 mg/d between groups.				Not reported

S17	National Cancer Institute- (NCI-) Common Toxicity Criteria, Version 2.0 (vomiting, grades 0–4)^f^	Complete relief (vomiting grade 0) **Day 1: acute vomiting** I = 18 (75.00%), C = 16 (66.67%) **Day 2–7: delayed vomiting** Day 2: I = 18 (69.23%), C = 10 (41.67%) Day 3: I = 17 (65.38%), C = 9 (37.50%) Day 4: I = 15 (57.69%), C = 7 (29.17%) Day 5: I = 16 (61.54%), C = 8 (33.33%) Day 6: I = 19 (73.08%), C = 11 (45.83%) Day 7: I = 25 (96.15%), C = 18 (75.00%)	Partial relief (vomiting grade 1) **Day 1: acute Vomiting** I = 3 (12.50%), C = 4 (16.67%) **Day 2–7: delayed Vomiting** Day 2: I = 5 (19.23%), C = 8 (33.33%) Day 3: I = 5 (19.23%), C = 10 (41.67%) Day 4: I = 8 (30.77%), C = 15 (62.50%) Day 5: I = 8 (30.77%), C = 15 (62.50%) Day 6: I = 6 (23.08%), C = 13 (54.17%) Day 7: I = 1 (3.85%), C = 6 (25.00%)	Minor relief + failure (vomiting grades 2–4) **Day 1: acute Vomiting** I = 3 (12.50%), C = 4 (16.67%) **Day 2–7: delayed Vomiting** Day 2: I = 3 (11.54%), C = 6 (25.00%) Day 3: I = 4 (15.38%), C = 5 (20.83%) Day 4: I = 3 (11.54%), C = 2 (8.33%) Day 5: I = 2 (7.69%), C = 1 (4.17%) Day 6: I = 1 (3.85%), C = 0 (00.00%) Day 7: I = 0 (00.00%), C = 0 (00.00%)	**Day 1: acute vomiting** I = 87.50%, C = 83.33% **Day 2–7: delayed vomiting** Day 2: I = 88.46%, C = 75.00% Day 3: I = 84.62%, C = 79.17% Day 4: I = 88.46%, C = 91.67% Day 5: I = 92.31%, C = 95.83% Day 6: I = 96.15%, C = 100.00% Day 7: I = 100.00%, C = 100.00%	No adverse events

S18	WHO Recommendations for Grading of Acute and Subacute Toxicity (nausea/vomiting, grades 0-4)^g^	Grade 0 **Acute CINV** I = 19 (63.33%), C = 14 (58.33%) **Delayed CINV** I = 18 (60.00%), C = 8 (33.33%)	Grade 1 **Acute CINV** I = 9 (30.00%), C = 8 (33.33%) **Delayed CINV** I = 9 (30.00%), C = 6 (25.00%)	Grades 2–4 (Minor relief + no effect) **Acute CINV** I = 2 (6.67%), C = 2 (8.33%) **Delayed CINV** I = 3 (10.00%), C = 10 (41.67%)	**Acute CINV** I = 93.33%, C = 91.67 **Delayed CINV** I = 90.00%, C = 58.33%	Not reported

S19	WHO Recommendations for Grading of Acute and Subacute Toxicity (nausea/vomiting, grades 0–4)	Grades 0-1 I = 32 (88.89%), C = 11 (35.48%)	Grade 2 I = 3 (8.33%), C = 12 (38.71%)	Grades 3-4 I = 1 (2.78%), C = 8 (25.81%)	I = 97.22%, C = 74.19%	Not reported

S20	WHO Recommendations for Grading of Acute and Subacute Toxicity (nausea/vomiting, grades 0–4)^h^	Grades 0-1 I = 46 (65.71%), C1 = 33 (66.00%), C2 = 15 (28.30%)	Grade 2 I = 14 (20.00%), C1 = 11 (22.00%), C2 = 18 (33.96%)	Grade 3 I = 10 (14.29%), C1 = 6 (12.00%), C2 = 20 (37.74%)	I = 85.71%, C1 = 88.00%, C2 = 62.26%	Not reported

S21	WHO Recommendations for Grading of Acute and Subacute Toxicity (nausea/vomiting, grades 0–4)^h^	Grades 0-1 I = 16 (40.00%), C = 7 (17.50%)	Grade 2 I = 18 (45.00%), C = 10 (25.00%)	Grade 3 I = 6 (15.00%), C = 23 (57.50%)	I = 85.00%, C = 42.50%	Not reported

I: intervention group, C: control group, CINV: chemotherapy-induced nausea and vomiting, WHO: World Health Organization, and NA: not applicable.

^a^Study.

^b^WHO Recommendations for Grading of Acute and Subacute Toxicity (nausea/vomiting, grades 0–4)^*^, Grade 0: none, Grade 1: nausea, Grade 2: transient vomiting, Grade 3: vomiting requiring therapy, and Grade 4: intractable vomiting [45].

^c^Morrow Assessment of Nausea and Emetics: A 17-item self-rated questionnaire used for assessing the occurrence, duration, and severity of nausea and vomiting.

^d^Diagnostic Standard for TCM Differentiation of Symptoms and Signs, A standardized diagnostic criteria developed by State Administration of TCM of People's Republic of China.

^e^Therapeutic effects of auricular therapy on adverse events caused By antiemetic medications.

^f^National Cancer Institute-Common Toxicity Criteria, Version 2.0: vomiting, grade 0: none, grade 1: 1 episode in 24 hours over pretreatment, grade 2: 2–5 episodes in 24 hours over pretreatment, grade 3: ≥6 episodes in 24 hours over pretreatment or need for IV fluids, grade 4: requiring parenteral nutrition, or physiologic consequences requiring intensive care, hemodynamic collapse [46].

^g^Used the WHO Recommendations to judge the incidence and severity of nausea and vomiting but did not specify the source of the criteria.

^h^Only applied the first four grades (grades 0–3) to evaluate the incidence and severity of nausea and vomiting.
